# Spatio-Temporal Analysis of Infectious Diseases

**DOI:** 10.3390/ijerph16040669

**Published:** 2019-02-25

**Authors:** Antonio López-Quílez

**Affiliations:** Department of Statistics and Operations Research, Faculty of Mathematics, University of Valencia, 46100 Valencia, Spain; antonio.lopez@uv.es

Epidemiological research on the pathogenesis, diagnosis, and treatment of infectious diseases is a broad field of study with renewed validity in the face of social changes and new threats. The spatio-temporal distribution of diseases is central to the knowledge of their development, transmission, spread, and dynamics.

New technologies and geographic information system (GIS) analysis together with highly structured mathematical and statistical techniques have a special utility in describing and analyzing the incidence of infectious diseases. Specifically, Bayesian inference methods allow the analysis of models with complex and flexible structures suitable to represent the diverse characteristics present in each geographical environment and disease.

Tuberculosis, hepatitis, human immunodeficiency virus (HIV), influenza, malaria, dengue, zika, and other vector-borne diseases are a constant concern for health authorities, practitioners, and patients. A variety of environmental, climatic, and socio-economic factors underlie their spatio-temporal patterns. In addition, factors such as changes in climate, habits, or land use intervene and complicate the understanding of these processes.

This Special Issue compiles contributions on the spatio-temporal analysis of infectious diseases and related themes. The collection of studies presented in this Special Issue contributes to a better understanding of which methods are currently available related with space and time in the data analysis of infectious diseases. These articles provide novel insights into the interaction between space and time in the context of each specific infectious disease, providing new perspectives in the understanding of these pathologies and their extent. A total of 11 manuscripts [1,2,3,4,5,6,7,8,9,10,11] were accepted after a single-blind review process by at least two international experts using the journal-specific review guidelines.

The different works corresponding to these articles were carried out in different geographical areas of several countries: China [2,5,6,7,8,10], Colombia [4], Ecuador [3], Korea [9], and Taiwan [1]. One of the studies deals with the analysis of an intracellular geometric model [11].

This volume contains a blend of papers focusing on the modeling, inference, and prediction of the behavior of infectious diseases. The scope of applications covers a wide range of topics. Several infectious diseases are addressed by studying their differentiated characteristics and relating them to relevant explanatory variables. The list of diseases studied includes typhoid and paratyphoid fevers [10]; Middle East respiratory syndrome-related coronavirus (MERS-CoV) [9]; tuberculosis [8]; bacillary dysentery [7]; cystic echinococcosis [6]; hand, foot, and mouth disease [5]; zika virus disease (ZVD) [4]; dengue [1,3,4]; and hepatitis [2,11].

While the contributions collected in this Special Issue are encouraging, it is evident that more discussion and research is needed to improve our understanding of the complexity of modeling infectious diseases in the space-time context. I hope that this Special Issue will stimulate and promote new action and research on this important topic.

## Abbreviations

The following abbreviations are used in this manuscript:

GIS	Geographic information system
HIV	Human immunodeficiency virus
MERS-CoV	Middle East respiratory syndrome-related coronavirus
ZVD	Zika virus disease
